# Some at Risk for COVID-19 Are Reluctant to Take Precautions, but Others Are Not: A Case From Rural in Southern Iran

**DOI:** 10.3389/fpubh.2020.562300

**Published:** 2020-11-16

**Authors:** Masoud Yazdanpanah, Bijan Abadi, Nadejda Komendantova, Tahereh Zobeidi, Stefan Sieber

**Affiliations:** ^1^Department of Agricultural Extension and Education, Agricultural Sciences and Natural Resources University of Khuzestan, Khuzestan, Iran; ^2^Department of Biosystem Engineering, University of Maragheh, Maragheh, Iran; ^3^Advanced Systems Analysis (ASA) Program, International Institute for Applied Systems Analysis (IIASA), Laxenburg, Austria; ^4^Institute for Environmental Decisions, ETH Zurich, Zurich, Switzerland; ^5^Department of Agricultural Extension, Communication and Rural Development, University of Zanjan, Zanjan, Iran; ^6^Research Area 2 “Land Use and Governance”, Working Group: Sustainable Land Use in Developing Countries, Leibniz Centre for Agricultural Landscape Research (ZALF), Muncheberg, Germany; ^7^Department of Agricultural Economics, Faculty of Life Sciences, Thaer-Institute, Humboldt-Universität zu Berlin, Berlin, Germany

**Keywords:** perceived severity, perceived vulnerability, intention, behavior, protection motivation model

## Abstract

Little is known about the evaluative and cognitive foundations for adopting preventive measures to reduce the spread of COVID-19. Recognizing the existence of a gap in the knowledge describing the intention and behavior of participating in health measures, this study investigated the drivers that contribute to the intention to take health protective measures among 305 rural youth from the Dashtestan Region, Bushehr Province, and southern Iran, reached through an online survey. Protection motivation theory (PMT) served as the theoretical framework for the study. It was able to forecast variation in intentions and behaviors with accuracies of 39 and 64%, respectively. Furthermore, the variables of response efficiency, perceived severity, and self-efficacy had a positive and significant effect on protective intentions. Additionally, perceived severity, self-efficacy, and intention produced a positive and significant impression on behaviors, with most of the behavioral variance being accounted for by intention, as was hypothesized. In conclusion, it is suggested that health development including training measures that take account of both the concrete issues of health resources and technologies and of more abstract ones, such as mindset readiness, are important for engagement in positive health care behaviors. Accordingly, training-based interventions for rural youth should be contemplated, with the object of changing their intentions.

## Introduction

Recently, leaders in important components and functions of world societies, such as economics, social interactions, health, education, and politics, have been forced to grapple with COVID-19, occasionally in contexts that produce promising news and sometimes with outcomes that exacerbate conditions. In early December 2019, COVID-19, a new form of severe respiratory syndrome, appeared in Wuhan, Hubei Province, China (1). Since that time, approximately 30 million cases of viral infection and a significant number of deaths have been reported throughout the world (2). On January 30, 2020, the World Health Organization (WHO) called the disease's spread a pandemic and announced a global emergency (1).

Most studies of COVID-19 have focused on the medical and technical aspects of the subject, such as the causative agent of the disease and its pathogenesis, epidemiology, diagnosis, and treatment, along with possible preventive interventions (3, 4). These interventions are generally intended for use by urban residents. Although they are a substantial portion of the human population, actions targeting city dwellers leave people in rural areas out of the account, particularly their perception of the interventions. Young villagers are vital stakeholders because they are in direct contact with food resources in supply chains. Their travel to urban areas and remaining there in opposition to health advisories can result in the failure of interventions and can increase the spread of the disease. For this reason, the protection of this group and the encouragement of healthy behaviors within it are of major importance. The study of health protection measures in Iran is important because it was the first low- or middle-income country to suffer a major outbreak including rural areas, and learning from Iran's experience will help all low- and middle-income countries (5).

Using the evaluative–cognitive framework of protection motivation theory (PMT), this study paves the way to investigating the drivers through which protective intentions are established and that can serve as immediate triggers to prompt action to diminish outbreaks of COVID-19. The objectives of this study were to assess the predictive power of PMT, describe the drivers of behavioral intention in this context, and develop determinants for protective behaviors.

### Theoretical Framework

PMT, a well-known and widely used theory in social psychology and health studies, was first proposed by Rogers (6). This theory describes the factors that prompt people to perform or fail to perform a given health behavior (7). In this context, three factors can effectuate fear appeals: the chance that an event will be dangerous, the probability of this event, and the efficacy of the response. Each of these communication variables requires an evaluative cognition process that can change attitudes (6).

**Figure 1:**
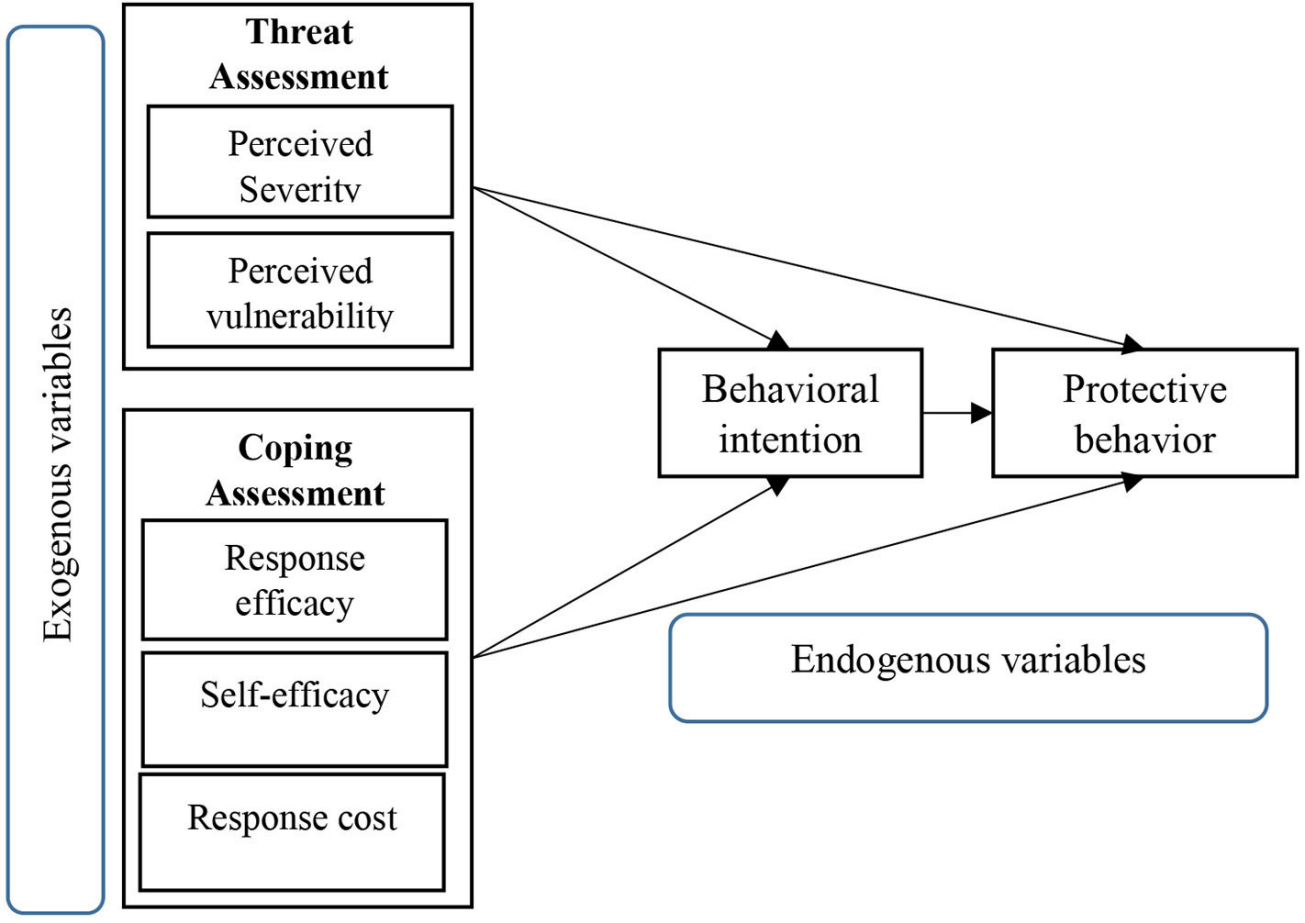
Protection motivation theory (6).

Two general and seminal ingredients in the PMT are subset constructs called threat and coping assessments (see Figure 1). A threat assessment is conceptualized by the two sub-concepts of perceived severity and perceived vulnerability, where the former refers to individuals' assessment of the negative consequences of a threatening security event (8–11), and the latter describes the extent to which one is likely to be respond to a health danger.

Coping assessment consists of response efficacy, cost, and self-efficacy (Figure 1). Response efficacy depends on the individual's belief in the effectiveness of a recommended behavior in reducing or eliminating the health threat (12). Perceived self-efficacy is defined as the person's belief in his or her competence to abide by the recommended behaviors and perform the necessary actions, along with obtaining desired results (13–16). Perceived costs include monetary, temporal, and cognitive costs, which are allocated to prevent the threat of a successful threat to a person's health (8). The PMT posits that the perception of the severity and vulnerability of a certain health threat contributes to discerning a perception of risk regarding it (6).

(5) found that the perceived severity of COVID-19 has a positive and significant association with behavioral intention. Díaz et al. (17) showed that perceived vulnerability to disease is connected with the fear of becoming contaminated through a disease vector. Helmes (7) provided evidence that PMT can predict 51% of the variance of a latent variable, response efficacy, where it is negatively associated with the motivation. Conversely, (5) reported that response efficacy is positively and significantly correlated with behavioral intention.

In the context of preventive behavioral intentions regarding MERS, Yoo et al. (18) established that self-efficacy has a significant and positive influence on handwashing and cough etiquette intentions. Self-efficacy has been found to be positively and significantly associated with the behavioral intention (5). Furthermore, Helmes (7) provided evidence that the response cost is positively correlated with motivation.

## Methods and Materials

This online cross-sectional survey was carried out in the Dashtestan Region, Bushehr Province, in southern Iran. The complete rural youth population (aged between 15 and 30 years old) of the research site was the research population. We used a self-developed, internet-distributed questionnaire that provided items describing behavior (eight items), behavioral intention (four items), response efficacy (five items), self-efficacy (two items), response cost (four items), perceived vulnerability (four items), and perceived severity (five items). The research items are presented in Table 1. We used a 5-point Likert scale for responses, from 1, “very low,” to 5, “very high.” The facial and content validity and psychometric properties of the questionnaire were confirmed by faculty members. The respondents stated that the questionnaire was clear and easy to complete, but in some cases, terms were used to clarify the items to allow them to better represent the variable being questioned.

**Table 1:** Concepts, statements, and reliability measured using Cronbach's alpha.

**Concepts**	**Items**	**X ± SD)**	**Cronbach's alpha**
Perceived severity	How likely do you think you are to get COVID-19 if you…	(3.40 ± 1.06)	0.89
	go out shopping?		
	go out to work or study?		
	go Out To Meet Your Relatives Or Friends?		
	leave home for any other purpose?		
Perceived vulnerability	To what extent will it…	(4.18 ± 0.71)	0.79
	be dangerous for you if you get COVID-19?		
	be costly for you if you get COVID-19?		
	affect your life if you get COVID-19?		
	affect your family if you get COVID-19?		
	affect your study if you get COVID-19?		
Response efficacy	The use of preventive measures and protective devices.	(4.06 ± 0.65)	0.71
	prevents the transmission of COVID-19.		
	prevents an outbreak of COVID-19 in the village.		
	has no effective consequences.^*^		
	does not affect the outbreak of COVID-19.^*^		
	prevents costly of treatment.		
Self-efficacy	If I want to, I could use preventive measures and protective devices.	(3.52 ± 0.75)	0.76
	The use of preventive measures and protective devices is relevant only to myself.		
Perceived cost	The use of preventive measures and protective devices is …	(2.90 ± 0.79)	0.66
	not worth it due to the cost.		
	expensive and costly.		
	difficult and laborious.		
Behavioral intention	I want to use COVID-19 protection measures and devices.	(4.28 ± 0.79)	0.91
	I intend to use COVID-19 protection measures and devices.		
	I plan to use COVID-19 protection measures and devices.		
	I encourage my friends and relatives to use COVID-19 protection measures and devices.		
Protective behavior	I stay home as much as possible and I do not go out	(4.17 ± 0.80)	0.86
	I wear a mask if I go out.		
	If I go out, I wear gloves.		
	I do not shake hands with people.		
	I regularly use disinfectant to disinfect my hands.		
	I regularly wash my hands with soap and water.		
	I wash and disinfect the materials I bring home from purchases.		
	I do not go to crowded and dangerous places so far as possible.		

(*) *Statements marked with asterisks were reverse coded*.

Internal reliability was confirmed between the measurement items for the research construct. The results showed that all values for composite reliability were above the minimum threshold of 0.70, ranging from 0.762 to 0.913. That is, all multiple-item measures for variables featured a satisfactory level of reliability (19). In the next step we considered the AVE values. As presented in Table 2, all AVE values for the research constructs surpassed the cutoff point of 0.50 (20). However, (20) showed that if the composite reliability is >0.6, an AVE of <0.5 is acceptable. The values ranged from 0.442 to 0.724. This indicated both convergent and discriminant validity. Furthermore, multicollinearity was checked by a correlation between the PMT constructs (Table 2). Neither bivariate correlation, however, exceeded the critical 0.70 thresholds (21), which is a robust sign that multi-collinearity problems were absent. Moreover, multi-collinearity problems were evaluated by assessing the tolerance (range = 0.62–0.88) and VIF (range = 1.12–1.60) scores, which fell within acceptable ranges. The acceptable range for Durbin–Watson values is between 1.5 and 2.5, and in this study, it was equal to 1.89, within that range, which indicates that there was no problem of multicollinearity. SPSS version 24 and AMOS version 20 were also used to analyze the dataset.

**Table 2:** The Pearson correlation test between all variables.

**Variables**	**1**	**2**	**3**	**4**	**5**	**6**	**7**
1. Perceived severity	1						
2. Perceived vulnerability	0.53^**^	1					
3. Response efficacy	0.25^**^	0.25^**^	1				
4. Self-efficacy	0.12^*^	0.14^*^	0.16^*^	1			
5. Response costs	−0.09	−0.24^**^	0.29^**^	−0.09	1		
6. Intention	0.28^**^	0.37^**^	0.43^**^	0.33^**^	−0.08	1	
7. Protective behavior	0.30^**^	0.38^**^	0.39^**^	0.29^**^	−0.04	0.69^**^	1
CR	0.808	0.889	0.762	0.763	0.804	0.913	0.863
AVE	0.461	0.618	0.457	0.617	0.673	0.724	0.442
Goodness-of-fit statistics:	Chi square = 563.097, Df = 354, Relative Chi-Sq = 1.591, AGFI = 0.832, GFI = 0.863, CFI = 0.952, IFI = 0.953, RMSEA = 0.044						

** p < 0.01 and

* *p < 0.05*.

## Results

### Descriptive Statistics

The mean age of the respondents to the study was 24.79 years. The youngest respondent was 15 years old, and the oldest was 30 years old. In the complete set of respondents, 125 people (41%) were male, and 180 were female (59%). The average household size was 4.68, with a standard deviation of 1.67 and a range from 1 to 16.

### Inferential Statistics

#### Correlation Between the Research Variables

The Pearson correlation coefficient was used to indicate the association between independent variables and protective behavior, the dependent variable. As shown in Table 2, protective behavior is positively and significantly correlated with perceived severity (*r* = 0.30, *p* < 0.01), perceived vulnerability (*r* = 0.38, *p* < 0.01), response efficacy (*r* = 0.39, *p* < 0.01), self-efficacy (*r* = 0.29, *p* < 0.01), and behavioral intention (*r* = 0.69, *p* < 0.01). These results indicate that there is not a high correlation among the independent variables.

Structural equation modeling is an appropriate and commonly used multivariate approach and was used to develop the structure of the conceptual model. As shown in Table 3, the results of the fit indices were compared to the standard cutoff measures to indicate the fit of the conceptual model to the dataset in a tailored manner.

**Table 3:** Assessment of the overall fit measurement of the SEM.

	**Indexes**	**RMSEA**	**CMIN/DF**	**CFI**	**NFI**	**IFI**	**GFI**	**AGFI**
Fit indices	Cutoff thresholds	≤0.08	≤3	0.9≤	0.9≤	0.9≤	0.9≤	0.9≤
	PMT	0.044	1.591	0.952	0.882	0.953	0.888	0.863

**Figure 2:**
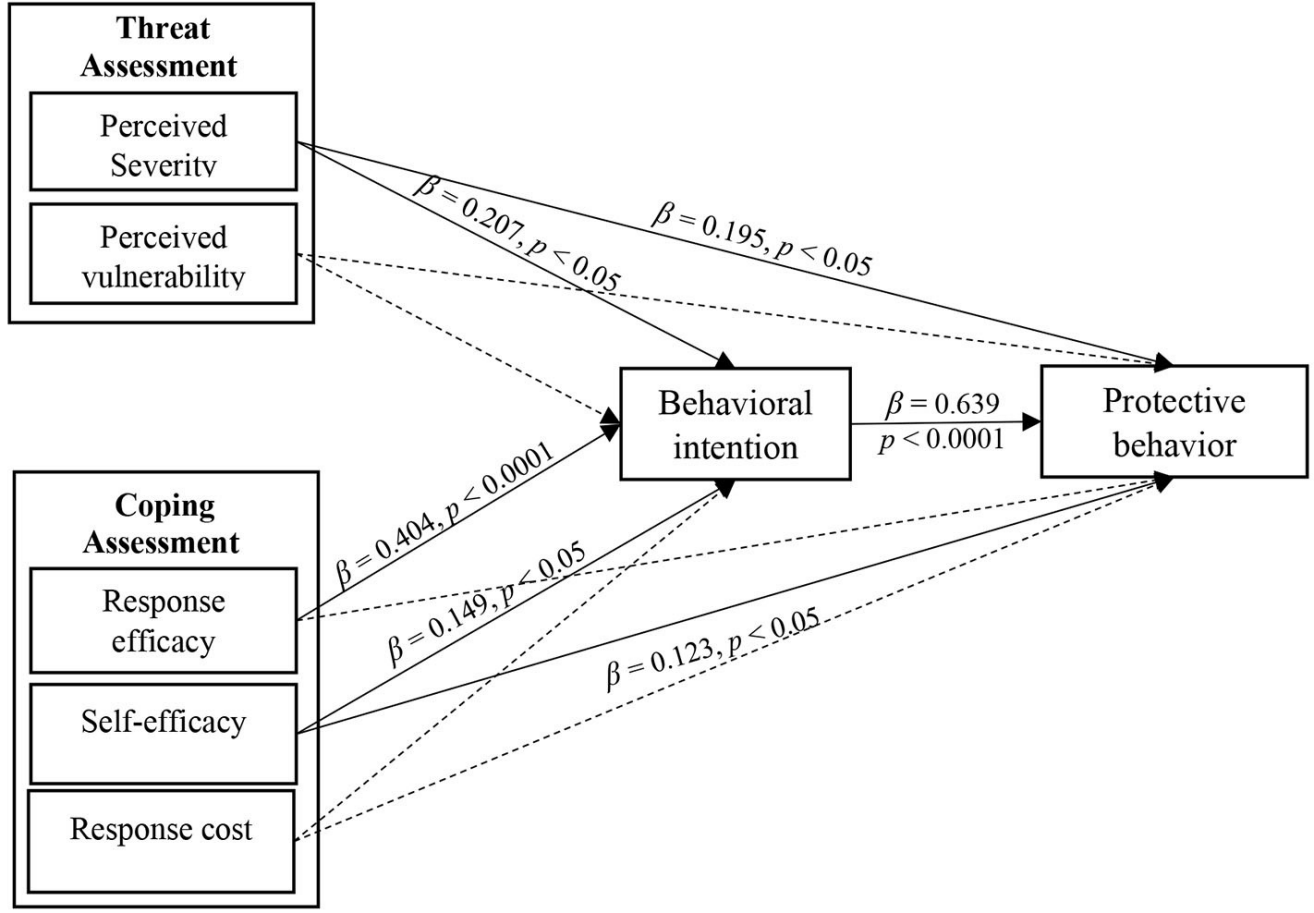
Structural equations modeling and path coefficients.

We gained insight into the robust power of the PMT to predict variation in behavior, which was 64%. We also obtained the following observations with respect to the effects of exogenous variables on the behavioral intentions and protective behaviors of rural youth against COVID-19. As shown in Figure 2, it was found that perceived severity (β = 0.207, *p* < 0.05), response efficacy (β = 0.404, *p* < 0.0001), and perceived self-efficacy (β = 0.149, *p* < 0.05) have a positive and significant impact on protective intention, and a considerable share of the prediction relates to the response efficiency variable. In total, these variables predicted 39% of variation in protective intention. Moreover, perceived vulnerability and perceived cost had no significant impact on behavioral intention.

The variables of perceived severity (β = 0.195, *p* < 0.05), perceived self-efficacy (β = 0.123, *p* < 0.05), and intention (β = 0.639, *p* < 0.0001) positively and significantly affected protective behavior, and among these variables, intention was the chief contributor to it. The variables of severity, self-efficacy, and intention together were able to predict 64% of the variation in the protective behavior, and perceived vulnerability and perceived cost had no significant impact on behavior. In addition, response efficacy (β = 0.259, *p* < 0.001), perceived severity (β = 0.132, *p* < 0.05), and self-efficacy (β = 0.095, *p* < 0.05) had significant indirect effects on protective behavior.

## Discussion

Perceived severity, an exogenous variable in the model, has an influence on behavioral intention. This shows that the respondents recognized that COVID-19 has a significant effect on health. Of course, part of this perception, as indicated by (22), is due to evaluative representations in memory, which includes experiences derived from altered living and occupational conditions owing to the disease. Furthermore, hearing of the number of people infected or killed and the impact of the disease on livelihoods, market outcomes, income, and human relationships all relate to people's overall experience. This finding is consistent with the results of (5). Perceived severity also directly affects protective behavior, which is also consistent with previous studies (23–26).

The influence of self-efficacy on behavioral intention is justified by the consideration that when respondents perceive that they have the ability to take preventive measures, they are expressing mental readiness to participate in coping behaviors. This finding is consistent with the work of Yoo et al. (18). Lee and Kang (27) showed that self-efficacy in patient care during an outbreak of infectious disease is the strongest predictor of patient care willingness.

Response efficacy had a significant effect on the willingness to engage in protective behaviors. Respondents perceived that activities and preventive measures are effective for treating COVID-19 disease. This suggests that the use of these measures can help improve health and return the social and economic conditions and even livelihoods to normality. This finding is consistent with those of (5), although their analyses were based on correlation, and in this study, structural modeling was used. Similarly, Camerini et al. (28) showed that understanding the effectiveness of vaccination response increased the desire for vaccination.

In addition, the effects of behavioral intention on preventive behaviors include being mentally ready and producing the mental willingness to perform preventive behaviors. Behavioral intention is the antecedent to behavior formation. The more that people follow health advice or plan to do so, the greater the occurrence of preventive behaviors. This finding is consistent with previous research (29).

The impact of self-efficacy on behavior suggests that respondents had the ability and skills and possessed the necessary environmental conditions to engage voluntarily in preventive measures. Respondents' support in terms of their livelihood, infrastructural, economic, and social dimensions produced enrichment of their intentions and behavior. Perceived self-efficacy showed a positive, direct, and significant effect on preventive behavior against COVID-19. The more motivated the respondents felt, the more capable and hopeful they were regarding success in fighting COVID-19 and the more protective behaviors they performed. Self-efficacy denotes the belief in one's own ability to perform a behavior. Individuals' behavior largely depends on the complexity and difficulty of a certain activity (self-efficacy) (30). Here, self-efficacy indicates the extent to which a person feels that he or she can perform protective and preventive practices against COVID-19. In other words, it indicates people's level of motivation and ability to observe healthy behaviors and prevent the spread of COVID-19. The easier it is for people to take preventive behaviors, the more prevention they will engage in. This result is consistent with those of previous studies (26, 31).

## Conclusion

This study investigated the determinants of intention and preventive behaviors of rural youth in the context of the COVID-19 pandemic to measure the power of PMT. It was found that the variables of response efficiency, perceived severity, and self-efficacy positively and significantly influenced intentions. Hence, it is suggested that incentive-training courses should be established by the health authorities to encourage rural youth to take protective measures, and the content of the training should be outlined in such a way that rural youth are exposed to the protective measures that have the most substantial potential to prevent the spread of the disease. This could be done by establishing classes in public places in rural areas. Perceived severity had a significant positive effect on behavioral intention and indicated how far rural youth understand the severity of the crisis. This finding can serve as a starting point for educational and technical initiatives that should be taken to educate rural youth due to the perceptual ground created regarding the severity of the disease. Another influential variable is self-efficacy, and the results for this factor indicated that rural youth have the necessary perceptions to enable them to enact sufficient protective measures. Thus, support for rural youth strengthens their perceived abilities and competencies, particularly in terms of financial resources, to help them cope with the COVID-19 pandemic and to enable them to make use of these measures.

## Data Availability Statement

The raw data supporting the conclusions of this article will be made available by the authors, without undue reservation.

## Ethics Statement

Ethical review and approval was not required for the study on human participants in accordance with the local legislation and institutional requirements. Written informed consent to participate in this study was provided by the participants' legal guardian/next of kin.

## Author Contributions

MY: conceptualization, methodology, software, formal analysis, investigation, writing—original draft, project administration, and funding acquisition. BA: validation, formal analysis, and writing—review and editing. NK: conceptualization, methodology, formal analysis, and writing—original draft. TZ: writing—review and editing. SS: conceptualization and writing—review and editing. All authors contributed to the article and approved the submitted version.

## Conflict of Interest

The authors declare that the research was conducted in the absence of any commercial or financial relationships that could be construed as a potential conflict of interest.
